# Supplementary material for: Periodic Boosters of COVID-19 Vaccines Do Not Affect the Safety and Efficacy of Immune Checkpoint Inhibitors for Advanced Non-Small Cell Lung Cancer: A Longitudinal Analysis of the Vax-On-Third Study
Source: Cancers (Basel). 2025 Jun 11;17(12):1948. doi: 10.3390/cancers17121948 (PMC12191419; doi:10.3390/cancers17121948)
Supplement: Supplementary file 1 [file cancers-17-01948-s001.zip › cancers-3647648-supplementary.pdf]

SUPPLEMENTARY MATERIAL

Title

Periodic boosters of COVID-19 vaccines do not affect the safety and efficacy of immune checkpoint inhibitors for advanced non-small cell lung cancer. A longitudinal analysis of the Vax-On-Third study.

Agnese Fabbri [1], Enzo Maria Ruggeri [1], Antonella Virtuoso [1], Diana Giannarelli [2], Armando Raso [3], Fabrizio Chegai [3], Daniele Remotti [4], Carlo Signorelli [1], and Fabrizio Nelli [1*]

[1] Department of Oncology and Hematology, Medical Oncology Unit, Central Hospital of Belcolle, Viterbo, Italy

[2] Biostatistics Unit, Scientific Directorate, Fondazione Policlinico Universitario A. Gemelli, IRCCS, Rome, Italy

[3] Department of Oncology and Hematology, Thoracic and Interventional Radiology, Central Hospital of Belcolle, Viterbo, Italy

[4] Department of Oncology and Hematology, Pathology Unit, Central Hospital of Belcolle, Viterbo, Italy

* **Correspondence:** Fabrizio Nelli, MD; Department of Oncology and Hematology, Medical Oncology Unit, Central Hospital of Belcolle, Strada Sammartinese snc, 01100 Viterbo, Italy, Phone +390761339055, Fax +390761339039, e-mail: fabrizio.nelli@asl.vt.it, ORCID iD: 0000-0001-8374-1362

Supplementary Table S1. Patient characteristics of PSM-unadjusted population

Variable	All patients (N=226)	Reference cohort (N=114)	Exposed cohort (N=112)	P value
Age<br>- mean (SD), years<br>- ≥70 years	 68.9 (8.3)<br>125 (55.3%)	 69.2 (7.8)<br>67 (58.8%)	 68.7 (8.8)<br>58 (51.8%)	 0.730<br>0.349
Sex<br>- female<br>- male	 68 (30.1%)<br>158 (69.9%)	 32 (28.1%)<br>82 (71.9%)	 36 (32.1%)<br>76 (67.9%)	0.563
ECOG PS<br>- 0<br>- 1<br>- 2	 64 (28.3%)<br>126 (55.8%)<br>36 (15.9%)	 41 (36.0%)<br>54 (47.4%)<br>19 (16.7%)	 23 (35.9%)<br>72 (64.3%)<br>17 (15.2%)	0.021
Histology<br>- non-squamous<br>- squamous	 165 (73.0%)<br>61 (27.0%)	 88 (77.2%)<br>26 (22.8%)	 77 (68.8%)<br>35 (31.3%)	0.178
Metastatic sites<br>- ≤2<br>- >2	 121 (53.5%)<br>105 (46.5%)	 57 (50.0%)<br>57 (50.0%)	 64 (57.1%)<br>48 (42.9%)	0.290
Bone metastasis	52 (23.0%)	28 (24.6%)	24 (21.4%)	0.637
CNS metastasis	48 (21.2%)	20 (17.5%)	28 (25.0%)	0.195
Liver metastasis	25 (11.1%)	14 (12.3%)	11 (9.8%)	0.673
PD-L1 TPS<br>- <1%<br>- ≥1% and ≤49%<br>- ≥50%	 72 (31.9%)<br>61 (27.0%)<br>93 (41.2%)	 36 (31.6%)<br>28 (24.6%)<br>50 (43.9%)	 36 (32.1%)<br>33 (29.5%)<br>43 (38.4%)	0.632
BMI<br>- mean (SD), kg/m^2<br>- ≥25 kg/m^2	 25.9 (4.4)<br>102 (45.1%)	 26.1 (4.9)<br>57 (50.0%)	 25.6 (3.9)<br>45 (40.2%)	 0.632<br>0.144
Smoking habits<br>- never<br>- current or former	 18 (8.0%)<br>208 (92.0%)	 8 (7.0%)<br>106 (93.0%)	 10 (8.9%)<br>102 (91.1%)	0.631
Previous thoracic RT	35 (15.5%)	12 (10.5%)	23 (20.5%)	0.044
LIPI category<br>- 0<br>- 1<br>- 2	 86 (38.1%)<br>88 (38.9%)<br>52 (23.0%)	 46 (40.4%)<br>38 (33.3%)<br>30 (26.3%)	 40 (35.7%)<br>50 (44.6%)<br>22 (19.6%)	0.195
Upfront therapy<br>- only ICIs<br>- pemetrexed-based<br>- paclitaxel-based	 93 (41.2%)<br>96 (42.5%)<br>37 (16.4%)	 50 (43.9%)<br>50 (43.9%)<br>14 (12.3%)	 43 (38.4%)<br>46 (41.1%)<br>23 (20.5%)	0.239
Corticosteroid therapy [a]	94 (41.6%)	41 (36.0%)	53 (47.3%)	0.105
APAP [b]	87 (38.5%)	42 (36.8%)	45 (40.2%)	0.682
Systemic antimicrobial therapy [c]	54 (23.9%)	29 (25.4%)	25 (22.3%)	0.641
PPI [d]	82 (36.3%)	44 (38.6%)	38 (33.69%)	0.491

PSM, propensity score matching; SD, standard deviation; ECOG PS, Eastern Cooperative Oncology Group Performance Status; CNS, central nervous system; PD-L1 TPS, programmed cell death ligand-1 tumor proportion score; BMI, body mass index; RT, radiotherapy; LIPI, lung immune prognostic index; APAP, acetaminophen; PPI, proton pump inhibitors. [a] exposure to high dose corticosteroid drugs (prednisone equivalent ≥10 mg daily for at least 5 days) within the 30 days prior to the start of treatment (not including premedication for chemotherapy); [b] exposure to therapeutic dose of APAP (at least 1000 mg per day for more than 24 hours) during the 30 days prior to the start of treatment; [c] exposure to therapeutic dose of any systemic antibiotics in the 30 days prior to the start of treatment; [d] exposure to any PPI dose at the start of treatment.

Supplementary Table S2. Multivariate analysis of immune-related adverse events in PSM-adjusted population (N=204)

Covariate	All grade irAEs		Grade 1-2 irAEs		Grade 3 irAEs	
	HR (95% CI)	P value	HR (95% CI)	P value	HR (95% CI)	P value
Age						
- <70 years	1.00	-	1.00	-	1.00	-
- ≥70 years	1.32 (0.63-2.78)	0.457	1.36 (0.61-3.02)	0.442	1.54 (0.31-7.47)	0.591
Sex						
- female	1.00	-	1.00	-	1.00	-
- male	2.68 (1.17-6.12)	0.019	1.82 (0.77-4.32)	0.172	3.70 (0.54-25.31)	0.182
ECOG PS						
- 0	1.00	-	1.00	-	1.00	-
- 1	0.90 (0.36-2.23)	0.833	1.07 (0.41-2.83)	0.880	0.63 (0.10-3.75)	0.620
- 2	1.38 (0.42-4.58)	0.590	2.36 (0.67-8.37)	0.181	0.21 (0.01-3.97)	0.304
Histology						
- non-squamous	1.00	-	1.00	-	1.00	-
- squamous	0.20 (0.05-0.71)	0.013	0.30 (0.08-1.05)	0.061	0.06 (0.01-4.98)	0.219
No. of metastatic sites						
- ≤2	1.00	-	1.00	-	1.00	-
- >2	0.54 (0.19-1.58)	0.267	0.74 (0.22-2.53)	0.639	0.27 (0.03-2.33)	0.237
Bone metastasis						
- none	1.00	-	1.00	-	1.00	-
- any	0.85 (0.31-2.29)	0.758	0.74 (0.23-2.33)	0.610	0.92 (0.15-5.36)	0.929
CNS metastasis						
- none	1.00	-	1.00	-	1.00	-
- any	1.59 (0.52-4.82)	0.409	1.07 (0.29-3.93)	0.909	4.48 (0.51-39.16)	0.175
Liver metastasis						
- none	1.00	-	1.00	-	1.00	-
- any	0.53 (0.12-2.36)	0.409	0.45 (0.08-2.58)	0.375	1.15 (0.07-16.82)	0.918
PD-L1 TPS						
-<1%	1.00	-	1.00	-	1.00	-
- ≥1% and ≤49%	1.56 (0.62-3.88)	0.339	1.78 (0.65-4.89)	0.257	0.90 (0.16-5.04)	0.907
- ≥50%	NA	0.999	10.46 (0.07-29.05)	0.717	NA	0.999

BMI<br>- <25 kg/m^2<br>- ≥25 kg/m^2	 1.00<br>0.52 (0.25-1.07)	 -<br>0.077	 1.00<br>0.69 (0.32-1.49)	 -<br>0.355	 1.00<br>0.12 (0.01-0.78)	 -<br>0.026
Smoking habits<br>- never<br>- current or former	 1.00<br>0.44 (0.12-1.63)	 -<br>0.225	 1.00<br>0.62 (0.15-2.53)	 -<br>0.515	 1.00<br>0.38 (0.02-7.10)	 -<br>0.524
Previous thoracic RT<br>- no<br>- yes	 1.00<br>0.51 (0.19-1.34)	 -<br>0.174	 1.00<br>0.54 (0.18-1.58)	 -<br>0.266	 1.00<br>0.33 (0.04-2.28)	 -<br>0.263
LIPI category<br>- 0<br>- 1<br>- 2	 1.00<br>0.31 (0.14-0.70)<br>0.27 (0.09-0.74)	 -<br>0.005<br>0.011	 1.00<br>0.38 (0.16-0.89)<br>0.19 (0.05-0.61)	 -<br>0.026<br>0.006	 1.00<br>0.27 (0.04-1.56)<br>0.85 (0.11-6.07)	 -<br>0.145<br>0.872
Upfront therapy<br>- only ICIs<br>- pemetrexed-based<br>- paclitaxel-based	 1.00<br>NA<br>NA	 -<br>0.999<br>0.999	 1.00<br>0.20 (0.01-12.52)<br>0.33 (0.01-21.80)	 -<br>0.453<br>0.610	 1.00<br>NA<br>NA	 -<br>0.999<br>0.999
Corticosteroids [a]<br>- no<br>- yes	 1.00<br>1.46 (0.66-3.25)	 -<br>0.346	 1.00<br>0.89 (0.36-2.16)	 -<br>0.801	 1.00<br>5.39 (0.95-30.39)	 -<br>0.056
APAP [b]<br>- no<br>- yes	 1.00<br>1.06 (0.51-2.18)	 -<br>0.865	 1.00<br>1.43 (0.66-3.08)	 -<br>0.355	 1.00<br>0.28 (0.04-1.61)	 -<br>0.155
Systemic antibiotics [c]<br>- no<br>- yes	 1.00<br>0.91 (0.37-2.23)	 -<br>0.837	 1.00<br>1.01 (0.37-2.73)	 -<br>0.148	 1.00<br>0.69 (0.10-4.66)	 -<br>0.703
PPI [d]<br>- no<br>- yes	 1.00<br>0.89 (0.41-1.92)	 -<br>0.767	 1.00<br>0.62 (0.26-1.45)	 -<br>0.271	 1.00<br>3.56 (0.75-16.92)	 -<br>0.110
Vaccine exposure<br>- no<br>- yes	 1.00<br>0.91 (0.43-1.91)	 -<br>0.812	 1.00<br>0.60 (0.27-1.37)	 -<br>0.233	 1.00<br>4.16 (0.81-21.33)	 -<br>0.087

irAEs, immune-related adverse events; HR, hazard ratio; CI, confidence interval; ECOG PS, Eastern Cooperative Oncology Group Performance Status; CNS, central nervous system; PD-L1 TPS, programmed cell death ligand-1 tumor proportion score; BMI, body mass index; RT, radiotherapy; LIPI, lung immune prognostic index; ICIs, immune checkpoint inhibitors; APAP, acetaminophen; PPI, proton pump inhibitors. [a] exposure to high dose corticosteroid therapy (prednisone equivalent ≥10 mg daily for at least 5 days) within the 30 days prior to the start of treatment (excluding premedication for chemotherapy); [b] exposure to therapeutic dose of APAP (at least 1000 mg per day for more than 24 hours) during the 30 days prior to the start of treatment; [c] exposure to any systemic antibiotics in the 30 days prior to the start of treatment; [d] exposure to any PPI dose at the start of treatment.

.
